# Synthesis of Nanoscale Liposomes via Low-Cost Microfluidic Systems

**DOI:** 10.3390/mi11121050

**Published:** 2020-11-28

**Authors:** Andres Aranguren, Carlos E. Torres, Carolina Muñoz-Camargo, Johann F. Osma, Juan C. Cruz

**Affiliations:** 1 Department of Electrical and Electronic Engineering, Universidad de los Andes, Cra. 1E No. 19a-40, Bogotá DC 111711, Colombia; a.aranguren@uniandes.edu.co; 2 Department of Biomedical Engineering, Universidad de los Andes, Cra. 1E No. 19a-40, Bogotá DC 111711, Colombia; ce.torres10@uniandes.edu.co (C.E.T.); c.munoz2016@uniandes.edu.co (C.M.-C.); 3 School of Chemical Engineering and Advanced Materials, The University of Adelaide, Adelaide, SA 5005, Australia

**Keywords:** liposomes, microfluidic, low-cost

## Abstract

We describe the manufacture of low-cost microfluidic systems to produce nanoscale liposomes with highly uniform size distributions (i.e., low polydispersity indexes (PDI)) and acceptable colloidal stability. This was achieved by exploiting a Y-junction device followed by a serpentine micromixer geometry to facilitate the diffusion between the mixing phases (i.e., continuous and dispersed) via advective processes. Two different geometries were studied. In the first one, the microchannels were engraved with a laser cutting machine on a polymethyl methacrylate (PMMA) sheet and covered with another PMMA sheet to form a two-layer device. In the second one, microchannels were not engraved but through-hole cut on a PMMA sheet and encased by a top and a bottom PMMA sheet to form a three-layer device. The devices were tested out by putting in contact lipids dissolved in alcohol as the dispersed phase and water as the continuous phase to self-assemble the liposomes. By fixing the total flow rate (TFR) and varying the flow rate ratio (FRR), we obtained most liposomes with average hydrodynamic diameters ranging from 188 ± 61 to 1312 ± 373 nm and 0.30 ± 0.09 PDI values. Such liposomes were obtained by changing the FRR from 5:1 to 2:1. Our results approached those obtained by conventional bulk synthesis methods such as a thin hydration bilayer and freeze-thaw, which produced liposomes with diameters ranging from 200 ± 38 to 250 ± 38 nm and 0.30 ± 0.05 PDI values. The produced liposomes might find several potential applications in the biomedical field, particularly in encapsulation and drug delivery.

## 1. Introduction

Microfluidic systems have gained considerable attention over the past decade due to their considerable potential for basic and applied scientific research in several fields, including organic synthesis, catalysis, fluid mechanics, thermodynamics, biotechnology, and medicine [1]. The most attractive feature of these systems is the possibility to manipulate small volumes of fluid inside microscale channels, which offers a few benefits such as low energy consumption as well as homogeneity of parameters, such as temperature and the possibility of continuous operation. Moreover, depending on the manufacturing technique, they may be produced at low costs [2]. Despite these benefits, most microfluidic experiments are conducted at low Reynolds numbers, where processes such as mixing are significantly limited due to the prevalence of the viscous forces [3].

Recently, the use of nanometer scale objects for the development of drug delivery carriers has propelled several applications in highly effective and specific pharmacological therapies [4]. One of the main challenges of this approach is, however, to assure that such objects comply with high quality standards in terms of repeatability and reproducibility of their physicochemical properties [3]. Conventional wet-chemistry techniques are generally difficult to adjust and control for producing nanoscale objects that exhibit parameters with very narrow size distributions. An alternative route is provided by microfluidic devices where the laminar flow conditions allow the controlled chemical synthesis or physical assembly of nanoscale objects [5,6]. In this regard, recent reports have demonstrated the efficient production of oxide nanoparticles, vesicles, polymeric capsules, and liposomes with highly uniform morphologies and size distributions [5]. Despite the growing interest in microfluidics, as evidenced by the increasing number of applications and types of devices, standardized protocols for manufacturing and characterization of nanoscale objects with the aid of these devices are rather scarce [5].

Liposomes are of particular interest for applications in biophysical studies and the screening of novel biomolecules with applications in therapeutics. These supramolecular structures were first produced by Bangham in the 1960s while studying phospholipids and blood clotting processes [7]. Liposomes are composed of lipid bilayers and are generally formed by self-assembly of phospholipids in aqueous buffers [5]. The assembly process is driven by the hydrophobic effect that organizes amphiphilic molecules by decreasing the entropically unfavorable interactions between the hydrophobic chains and the aqueous medium [7]. As a result, these vesicles are structured with a central aqueous compartment surrounded by one or more concentric phospholipid layers. Due to their resemblance with natural lipid bilayers present in cell membranes, liposomes can be successfully used as models to study biomolecular interactions [8]. Moreover, they can be employed as carriers to improve the therapeutic efficacy and half-life of drugs delivered to cells by increasing the drug internalization and absorption, while minimizing possible rapid degradation events, side effects, and toxicity [9]. Clinically, liposomal formulations have been exploited as carriers for the delivery of biological active molecules such as Doxorubicin, (glyco-) lipids, and siRNA [9,10,11]. This has enabled the development of a number of gene delivery, cancer treatment, and antifungal and anti-inflammatory therapies [9].

Various techniques have been proposed for the preparation of liposomes in which the dispersity, size, lamellarity, and volume of encapsulated materials have normally exhibited considerable variability [7,12]. Microfluidic systems have become a popular alternative to achieve a better control over the physical properties of synthesized liposomes [5,6]. In this case, the microfluidic device allows the possibility for a controlled mixing of two immiscible phases at the nanoliter scale under laminar conditions [6,13]. This can be achieved by the precise control of the Total Flow Ratio (TFR) and Flow Rate Ratio (FRR) of the streams containing the components for the self-assembly process [14]. As a result, liposomes of a uniform size and morphology are spontaneously formed as a discrete separated phase [15]. Depending on the need of an external energy source, the mixing processes could be either completely passive or active [16]. Besides the homogeneity of physical properties, the fine control over the conditions of the process provided by microfluidics is attractive for the large-scale and robust production of liposomes [17,18]. This is further supported by the reduced requirements of post-processing steps to polish and homogenize samples [19], and the possibility to prepare the liposomes and encapsulate the compounds of interest in one step with the same device [20,21]. Despite the tremendous potential of microfluidics, adjusting the operation conditions might require a considerable effort and extensive prototyping [22]. This poses a major limitation, as preparing the prototypes is likely to involve several steps as well as sophisticated and expensive cleanroom manufacturing techniques, such as bottom-up and top-down strategies [13,23]. To try to address this issue, recent reports have introduced much simpler and inexpensive fabrication schemes that include paper-based and laser cutting-based techniques [2,24,25,26]. Despite the invested efforts, rapid prototyping of devices with geometries representatives of those obtained with high-precision manufacturing techniques is still challenging.

Here, we aimed at putting forward laser cutting-based manufacturing techniques to assemble low-cost prototypes of microfluidic devices for the synthesis of the liposomes of uniform morphologies and size distributions. Moreover, we detailed the assembly process steps and the required materials and instruments for each step. We assembled two different devices with different levels of channel complexities to demonstrate the versatility of the manufacturing technique. The performance of the devices under various TFR and FRR values was also assessed in silico via multi-physics simulations in COMSOL Multiphysics^®^. The manufactured devices were successfully tested in the synthesis of unilamellar liposomes with average diameters of around 500 nm.

## 2. Materials and Methods

### 2.1. Materials

#### Reagents

Sodium chloride, anhydrous, free-flowing 746,398>99%, l-α-lecithin, soybean-cas 8002-43-5–calbiochem, Potassium chloride bioxtra p9333>99%, Potassium phosphate monobasic, anhydrous p0662>99%, Sodium phosphate dibasic, acs reagents9763>99%, and Chloroform c2432>99.5% were purchased from Merk (St. Louis, MO, USA). The 60 s. universal glue was from Loctite (Bogota, Colombia). Methyl methacrylate was acquired from Aquaterra S.A.S (Medellin, Colombia). 2-propanol (usp, bp, ph. eur.) pure, pharma grade > 99.5% was purchased from Illinois Tool Works (ITW) reagents (Bogota, Colombia).

### 2.2. Methods

In this study, we manufactured two microfluidic systems using Poly(methyl methacrylate) (PMMA) (Local distributors, Bogota, Colombia) sheets (3-mm thickness). The first one consisted of two PMMA sheets glued together with the bottom one engraved with a channel pattern (1-mm depth). The system was also equipped with a vertical outlet. The second one, comprised three PMMA layers glued together with the top and bottom layers serving as caps while the middle one had a cut through channel pattern at a depth of 3 mm. The first device is termed here as a double-layer while the second is termed as a triple-layer.

#### 2.2.1. Device Manufacture and Laser Cutting

**Figure 1:**
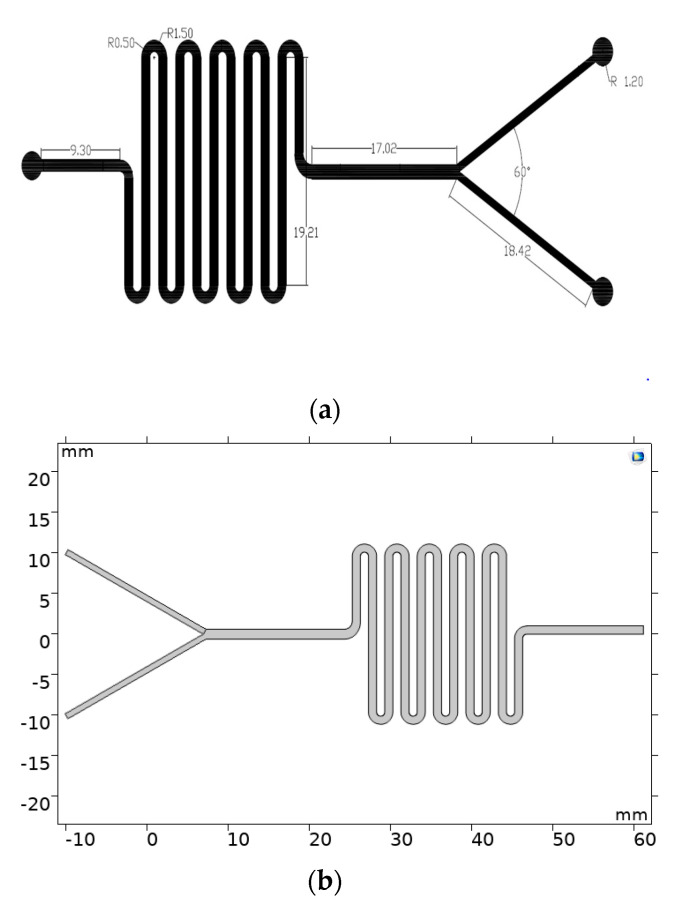
Design of geometries for manufacture and simulation of the microfluidic devices. (**a**) Microfluidic channel pattern designed for the two-layer device. The design was performed with the aid of AutoDesk^®^ 2019 (AutoCAD) software. Measurements are presented in millimeters and degrees. (**b**) 2D design implemented in COMSOL Multiphysics ^®^ for the finite elements simulations.

**Figure 2:**
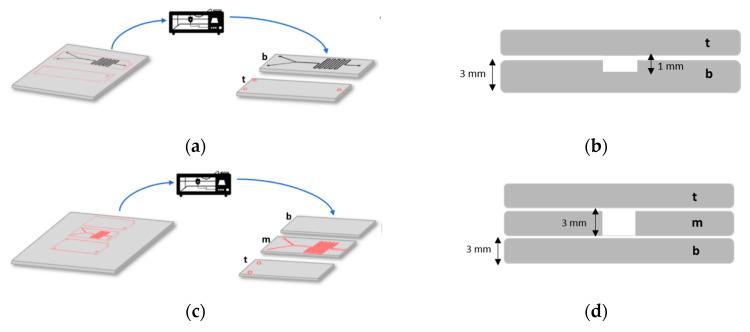
Microfluidic design pattern and laser cutting of device’s PMMA layer (t: top layer, m: middle layer, b: bottom layer). (**a**) Two-layers device. (**b**) Schematic of the cross-section view of the Two-layers device (channel width: 700 µm). (**c**) Three-layers device. (**d**) Schematic of the cross-section view of the Three-layers device (channel width: 700 µm).

Two rectangular sheets (width: 700 µm, height: 2.5 cm) were designed with the aid of AutoCAD (AutoDesk Inc., Mill Valley, CA, USA). One of them was then selected to insert the microfluidic channel pattern shown in Figure 1a. Next, the outer rectangular geometries were set in a red color, while the microfluidic pattern was set in black for further channel engraving. The engraving and cutting of the PMMA sheets (thickness:3 mm) was carried out using a laser cutter machine SPEEDY 100(60 W, TROTEC^®^, Marchtrenk, Upper Austria, Austria) at a fixed velocity (V) and power (P). For engraving, they were V = 0.85, and P = 90.00 while for cutting V = 7.18, and P = 54.20 (Figure 2). Finally, the PMMA debris was removed from the PMMA sheets by rinsing with an ethanolic solution (96% *v*/*v*) and blowing with an air pistol.

**Figure 3:**
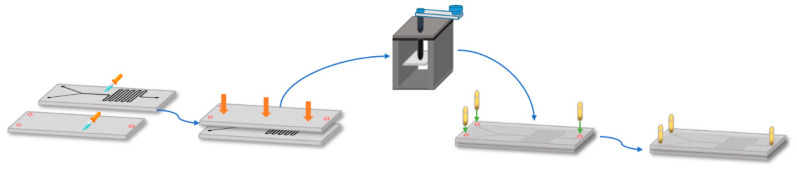
Assembly method for the two-layer device. Gluing PMMA sheets with the aid of 70% (*v*/*v*) ethanol and a mechanical press, which was followed by placement on a hot plate (110 °C).

**Figure 4:**
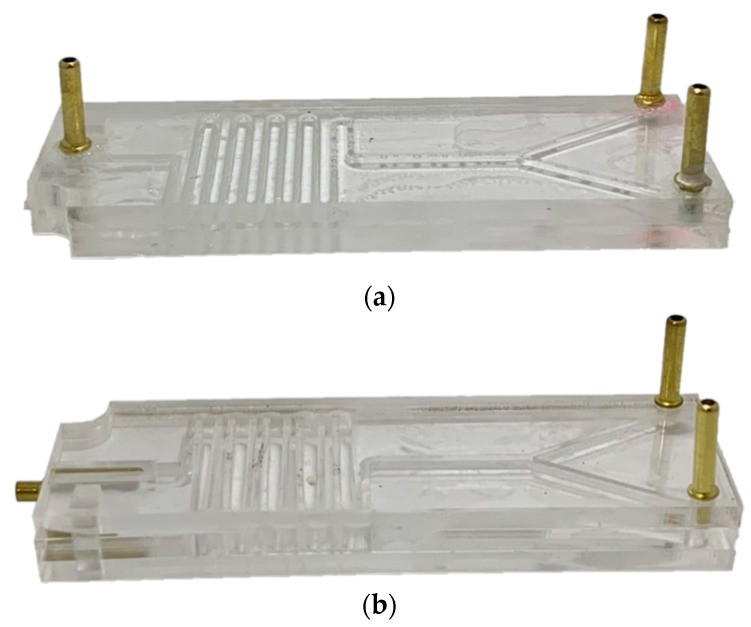
Actual pictures of the assembled devices. (**a**) Two-layers device. (**b**) Three-layers device.

The assembly started by putting together the PMMA sheets after spreading homogeneously (with a syringe), a 70% (*v*/*v*) ethanol solution on their surfaces. The two pieces are then maintained under a constant force for 10 min with the aid of a mechanical press, which was followed by placing the device on a hot plate (Temp: 110 °C). (Figure 3). Finally, three microfluidic connectors were attached to the top sheet of the device using a 60 s Universal Glue, which was subsequently left under room conditions for at least 12 h until the glue was fully dried (Figure 3). The assembled systems are shown in Figure 4. After assembly, the presence of leakages and channel blockages was evaluated using an infusion pump (Programmable touch screen syringe pump (78-8110c)) and a piece of infusion tubing (Nelaton, Probes, Medex caliber 8). Any detected leakage was fixed by adding a drop of methacrylate adhesive at the identified location and placing the device again in a hot plate (Temp: 110 °C). Finally, a digital universal serial bus (USB) microscope (UWT series, Amscope, Irvine, CA, USA) at a 10X magnification was used for the final inspection of the channel to identify clogs and debris residues, which may be pushed off the system during the purging stage prior to conducting the experiments (see below in the experimental setup section).

#### 2.2.2. Sample Preparation

To prepare the lipidic phase, 14 mL of Soy lecithin dissolved in isopropyl alcohol (IPA) solution was prepared at a concentration of 0.78 mg/mL and transferred to a 15-mL Falcon for further vortexing at 2900 RPM for 5 min. Additionally, the aqueous phase was either PBS (1X) or NaCl 0.05 M solutions.

#### 2.2.3. Multiphysics Simulations

The simulations were carried out in COMSOL Multiphysics^®^ 5.3 (COMSOL Inc., Stockholm, Sweden) to analyze the reciprocal diffusion between aqueous and lipidic phases along the mixing serpentine. This was accomplished by implementing the computational fluid dynamics (CFD) and the transport of diluted species modules. For the sake of saving time and computational resources, the finite element analysis was carried out on a 2D geometry produced with the COMSOL model builder, as is shown in Figure 1b. The first coupled physic in the model was the laminar flow, which describes the transport of momentum with the aid the Navier-Stokes equations for incompressible fluids (1) and the continuity of Equation (2).

(1) $$\nabla \left[-PI+\mu \left(\nabla u+{\left(\nabla u\right)}^{⊺}\right)\right]+F=0,$$

(2) ρ∇⋅(u)=0,

where P is the pressure, μ is the dynamic viscosity of the fluid, F is the volumetric forces, and ρ is the fluid density. The second coupled physic was the transport of diluted species, which relies on the convective–diffusive Equation (3) for the corresponding transport phenomena.

(3) $$\frac{\mathrm{dc}}{dt}+\nabla \cdot \left(-D\nabla C\right)+u\cdot \nabla c=R,\;$$

where c is the concentration of the lipid phase, u is the velocity field as calculated by the laminar flow module, D is the effective diffusivity of the lipid phase, and R is the net volumetric source for c. Additionally, an Inflow boundary condition was imposed at the end of the inlet channel as the initial concentration value at the Y-junction. Considering that in this section of the channel, the concentration begins to change. The equations for both physics were simultaneously solved in a time-dependent study with a step of 9 s using a mesh composed by 11,951 domain elements and 1615 boundary elements. The implemented solver was the multifrontal massively parallel sparse direct (MUMPS) mainly due to its optimized algorithm for fluid mechanic problems. All parameters for the simulation are listed in Table S1.

### 2.3. Experimental Setup

Three pieces of tubing of about a 20-cm length (Nelaton, Probes, Medex caliber 8) were cut and connected to the solutions’ inlets and the outlet of the system. This was to ensure that each tubing has a length of at least the distance from the syringe pumps to the microfluidic device to minimize dead volume. The tubing ends were cut at a 45° angle to facilitate insertion into the fluidic ports. Prior to using the system, a purging process of the channel was carried out with the aid of 10 mL of Isopropyl alcohol (IPA) and an infusion pump (Programmable touch screen syringe pump, 78-8110C, Vernon Hills, IL, USA) equipped with a 10-mL syringe and operating at a flow rate of 300 mL/min. To start the process, two 15-mL syringes were filled with the well-mixed lipid solution and the phosphate buffered saline (PBS) (1X) or NaCl solution and tilted and flicked vertically to move air bubbles to the syringe outlet.

The tubing previously attached to the fluidic ports was connected to the syringe’s outlets whose plungers were pushed manually in order to force the tubing’s dead volume until the fluid was about to enter the device. The syringes were securely mounted on an infusion pump (Programmable touch screen syringe pump (78-8110c)) and flow conditions were set considering a total flow rate (TFR) of 300 mL/h and flow rate ratios (FRR) (aqueous: solvent) ranging from 2:1 to 5:1. Finally, injection of the solutions was carried out sequentially, starting with the lipid solution until the first drop of liquid leaves the outlet and no bubbles were identifiable. This is followed by the injection of the aqueous solution (PBS or NaCl solutions). Considering empirical tests with varying FRR values of up to 5:1, we determined that this sequential order needed to be followed to ensure a continuous flow of both phases regardless of their flow rates differences. The obtained solutions were collected by locating the outlet tubing into a reservoir recipient after each solution had entered the device and the flow stabilized.

#### Characterization

The obtained liposome samples were characterized via a Zetasizer Nano ZS (Malvern, Panalytical, Egham, UK) to determine the size, polydispersity, and the zeta potential. Additionally, a transmission electron microscope (TEM) Tecnai F20 Super Twin TMP (FEI, Hillsboro, OR, USA) was used to observe their morphology and determine their size aided by the ImageJ software. For the preparation of the sample for TEM analysis, a drop of the sample was taken and deposited on a copper grid with carbon coating and allowed to dry for 1 hr. Subsequently, the samples were stained with 2% Uranyl Acetate by depositing one drop on the grid for 8 min. Finally, the grid was washed with deionized water and left to dry for later imaging at a total magnification of 29 kx.

### 2.4. Traditional Liposome Synthesis Methods

#### 2.4.1. Freeze Thaw-Method

A solution of egg lecithin in PBS (1X0.02 % (*w*/*v*) was added to a 0.05 M sodium chloride solution and vortexed for 3 min. The solution was then immersed in boiling water for 30 s, which was followed by cooling in an ice bath for 30 s. The process was repeated several times for 3 min. Finally, the sample was filtered through a 0.2-μm syringe filter (Thermoscientific, Waltham, MA, USA) thrice and analyzed using a Zetasizer Nano ZS (Malvern, Panalytical, Egham, UK) to determine the size, polydispersity, and the zeta potential of the obtained liposomes.

#### 2.4.2. Thin Film Hydration Method

A solution of 100 mg of soy lecithin dissolved in 10 mL of chloroform was evaporated in a Rotary evaporator Hei-VAP Value Digital Vertical (Heidolph, Schwabach, Germany) for 1 h at 45 °C under vacuum (50 mbar). Then, 20 mL of PBS (1X) were added to the rotavaporation flask and rotated for 45 more minutes at 55 °C and atmospheric pressure. Finally, the sample was filtered through a 0.2 μm syringe filter (Thermoscientific, Waltham, MA, USA) thrice and analyzed using a Zetasizer Nano ZS (Malvern, Panalytical, Egham, UK) to determine the size, polydispersity, and the zeta potential of the obtained liposomes.

## 3. Results and Discussion

As discussed below, two microfluidic devices were manufactured using 3-mm polymethyl methacrylate (PMMA) sheets. The first device consists of two PMMA layers on top of each other. The microchannel pattern was laser-engraved on the bottom layer to a depth of about 1 mm. The top layer was then glued with the aid of either alcohol or a methacrylate adhesive (see below for details). The vertical inlets and outlet were then equipped with hose connectors to pump the fluids. The second device was a three PMMA layer device where the top and bottom layers were glued to a central piece with the microchannel pattern cut through to a 3-mm depth. In this case, the fluid outlet is located horizontally along the plane of the channel. Both configurations were studied in detail experimentally and in silico with the intention of evaluating the impact of FRR on the properties and stability of the obtained liposomes.

### 3.1. Multiphysics Simulations

**Figure 5:**
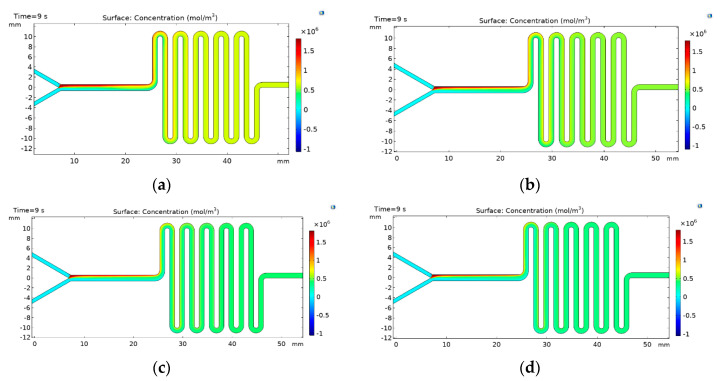
Concentration profiles for the lipidic phase within the channel in the two-layers device (**a**) FRR 2:1, (**b**) FRR 3:1, (**c**) FRR 4:1, and (**d**) FRR 5:1.

**Figure 6:**
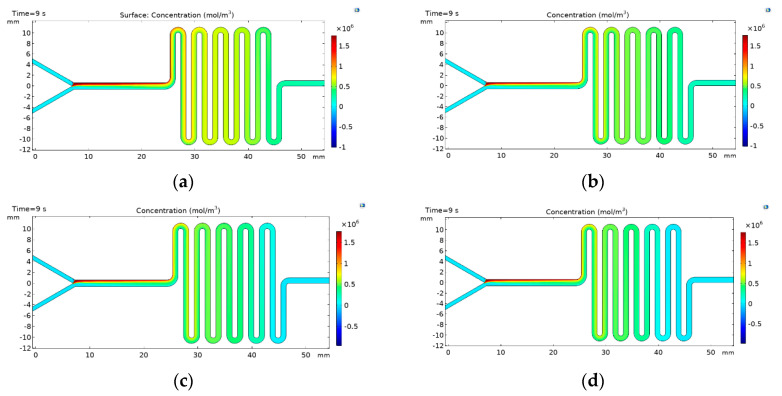
Concentration profiles for the lipidic phase within the channel in the three-layers device (**a**) FRR 2:1, (**b**) FRR 3:1, (**c**) FRR 4:1, and (**d**) FRR 5:1. Selection of flow rate and solvent to an aqueous flow rate ratio (FRR).

The liposomes self-assembly was evaluated by carrying out finite element simulations using COMSOL Mulitphysics^®^5.3 software (COMSOL Inc., Stockholm, Sweden). The simulation allowed us to understand the mixing level required to promote the appropriate phase exchanged for liposomes formation. Figure 5 shows the concentration profile results for the two-layers device for FRR values from 2:1 to 5:1. The results confirm that an increase in the inlet velocity difference between the aqueous phase and the lipid phase improves the mixing. These results also provide further evidence for the notion that liposomes formation and the reduction of the size is inversely proportional to the FRR, which correlates well with the observed improvement mixing [5,22,27]. Figure 6 shows the results for the three-layered device where similar trends are observed for the two-layers as a function of the FRR. Nevertheless, there is a strong difference in the mixing performance of this configuration that can be seen in the last section of the channel, where the concentration of the lipid phase is even lower when compared with the concentrations obtained using the two-layers device, thereby indicating a better phase exchange. This can be attributed to a slight increase in the Reynolds number caused by the increase in the channel depth [28]. The obtained results suggest that the designed devices provide the hydrodynamic conditions for sufficient mixing and, therefore, the opportunities for phase exchange and liposome formation.

### 3.2. FRR Characterization

Liposomes’ physical properties such as average size, polydispersity, and electric potential values have demonstrated a strong dependence on the FRR at which the microfluidic devices are operated during the synthesis process [22,29]. Accordingly, our study focused on evaluating the impact of varying the FRR on the performance of the designed devices. This was accomplished by altering the FRR between an aqueous solution, namely, phosphate buffered saline (PBS) or NaCl (0.05 M) and a solvent soy lecithin-based solution from 2:1 up to 5:1 while keeping a constant total flow rate (TFR) of 5 mL/min. This parameter is estimated as the sum of all streams entering the system, and appears to have a negligible impact on liposome physical characteristics [22]. Our experiments were conducted with aqueous buffers (i.e., PBS (1X) and NaCl (0.05 M)) due to their pH values close to neutrality (i.e., 7.5 and 7.4) and the possibility to implement the devices in the synthesis of liposomes for biomedical applications where such conditions are imperative. However, working under such conditions is challenging due to the limited colloidal stability of the liposomes, as shown by the low surface charge and the weak dependence of Zeta potential with the FRR (Table 1).

Table 1 summarizes the dynamic light scattering (DLS)results for the two-layer device shown in Figure S1. When using a PBS solution, liposomes exhibited a steady decrease from 250 nm to 188 nm since the FRR was increased. The polydispersity indexes (PDI) of the obtained liposomes were below 0.5 for the 3:1 to 5:1 FRRs, while those for the 2:1 FRR samples were above the base value. This increase in polydispersity may be possibly due to a reduced reciprocal diffusion between the solvent and aqueous phases, thereby increasing the chance of forming lipid aggregates. This, in turn, detrimentally decreases sample homogeneity. Since the FRR was increased, liposomes prepared in the presence of an aqueous phase enriched in NaCl also showed a steady decrease from 462 nm to 283 nm and PDI from 0.58 to 0.15, respectively. The fluctuations in the size observed for experiments at a 3:1 ratio can be attributed to small imperfections on the surface of the channels and their rapid fouling after only a few operating cycles. While this might be a limitation for continuous and large-scale operations, our devices still offer an inexpensive route for proof-of-concept experiments without significant investments in advanced manufacturing equipment.

Regarding the Zeta potential, samples obtained in the presence of NaCl solutions showed increasing values reaching a peak at −16.45 mV for the 5:1 ratio, while, for the PBS solution, Zeta potentials (mV) did not show a clear tendency.

DLS results for the three-layer device are shown in Figure S2 and summarized in Table 2. Unlike the two-layer configuration, in this case, the average sizes hovered between 220 nm and 340 nm. However, an outlier of 520 nm was shown for the 3:1 ratio, which can be attributed to not completely polished channels and fouling after a repetitive operation (see detailed discussion above). The PDI values were below 0.5 except for the 3:1 FRR. Liposomes obtained in NaCl solutions showed a significant size increase at FRRs of 3:1 and 4:1. Nonetheless, in both cases, the PDI values were well below 0.5, especially at the 5:1 ratio where it reached its lowest value at 0.14. Regarding the Zeta potential, none of the solutions exhibited a clear trend since the FRRs were increased. According to the results for both devices, the best samples considering their physical properties were obtained using PBS solution as a solvent at a 4:1 fixed FRR. Hence, it appears that the FRR and type of implemented aqueous solution are critical to control the average size and homogeneity of prepared liposomes. These parameters also have an impact on the mixing rate, which, in turn, is largely influenced by the device’s geometry.

In this regard, Y-junction geometries followed by serpentine or spiral-shaped geometries tend to induce chaotic advection along the channels [28] and establish parallel laminar flow streams of solvent and antisolvent that ultimately leads to diffusion at their interface [30]. This phase exchange leads to an increase in the polarity of the lipid solution to a critical point where liposomes are formed [6,13,31].

### 3.3. Colloidal Stability

Even though no clear tendencies were identified for zeta potentials as a function of FRR in either of the two configurations, the obtained values varied between −4 mV and −16 mV, which are comparable with the results reported for distearoylphosphatidylethanolamine-poly(ethyleneglycol)(DSPE-PEGF) liposomes where the zeta potential ranged from −8 mV to −13 mV [32]. Our results suggest, therefore, that the obtained liposomes exhibit only an acceptable colloidal stability considering the negligible impact of electrostatic interactions in preventing possible coalescence between liposomes [32,33,34]. For future medical applications such as drug delivery vehicles, liposomes’ colloidal stability may be improved by two mechanisms that increase repulsive forces. First, steric repulsion, which comprises the addition of polymers to the liposomes’ surface, generates a coating thick enough to maintain them separated by steric repulsion [35]. Second, electrostatic stabilization, which defines liposome interaction as a result of the distribution of charges in the aqueous dispersion medium, can be mainly determined by its ionic strength and pH [36]. Thus, if an alkaline solution is added to the suspension, the particles tend to acquire a more negative charge, whereas, by adding acidic solutions, the suspension will reach a point of neutrality. Further addition of acidic species will develop a positive charge on the surface. This can be easily visualized in the zeta potential vs. pH curve, where zeta potential will be positive at a low pH and negative at a high pH. The corresponding change of voltage sign is specified by the isoelectric point, where the zeta potential passes through zero [37].

The soybean-based lipid solutions implemented here have been reported to exhibit adequate size stability over time [38]. However, the relatively low colloidal stability observed suggests that other mechanisms might come into play and, for that reason, future work should be directed toward analyzing in detail such phenomena. Additionally, we propose to address stability issues over time by synthesizing the liposomes with other phospholipids such as DSPE and –PEGF or PEGylated versions of them.

### 3.4. Microscopy

**Figure 7:**
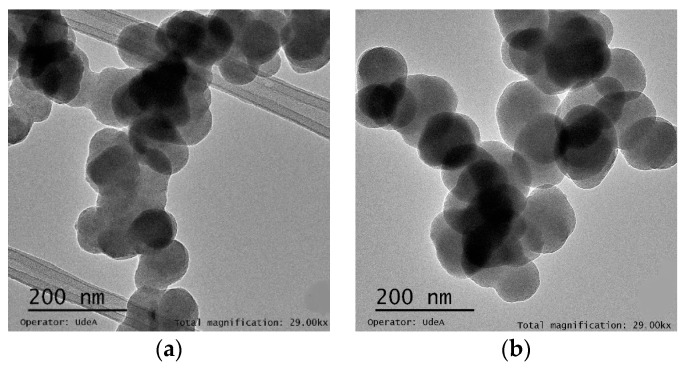
(**a**). TEM microscopy analysis of the liposomes sample prepared using the two-layer device, PBS solution at a 4:1 FRR ratio. (**b**). TEM microscopy analysis of liposomes prepared using the three-layer device, solution (0.05 M) at a 2:1 FRR ratio.

Figure 7 shows TEM microscopy images of the liposomes obtained with the two-layer and three-layer devices. The images confirm high liposomes density and homogeneity. For the first image, the average size found was approximately 108 ± 8 nm while, for the second, one was 135 ± 10 nm. These results are significantly different from those obtained via DLS analysis. As reported, DLS measures the hydrodynamic diameter of particles in a suspension [39] while TEM dimensions in a dried state that interact between them are not considered [40]. Nevertheless, these images confirm the adequate performance of the microfluidic device in the synthesis of nano-sized liposomes.

### 3.5. Comparison with Conventional Methods

To validate the microfluidic system performance in the synthesis of liposomes of high quality, we compared the prepared samples with those obtained via conventional and standardized Freeze-Thaw and Thin Film Hydration methods (TFH) (see Supplementary Figure S3 and the summarized results in Table 3). Despite the changes in size distribution from one batch to another (which can be addressed by a pretreatment of the channels’ surface [41,42]), our results indicate that the synthesized liposomes exhibit adequate average size, PDI, and acceptable zeta potential when compared with traditional methods. Compared with the TFH technique where the average size and the PDI achieved demonstrated the highest quality, there are several advantages offered by our microfluidic devices in terms of production time and post-processing stages. In the case of the production time, it only takes approximately 4 min with our microfluidic devices to obtain 20 mL of suspended liposomes with an approximate concentration of 1.5 mg/mL. In contrast, the TFH requires 2 h and 30 min to obtain the same volume without considering post-processing time to adjust size and distribution, which usually takes a few more hours. In this regard, the TFH technique generally relies on an extrusion-based method that has been reported as a widely used technique for sizing liposomes due to its simplicity, fast production, and effectiveness [43]. Despite these attractive features, this technique still exhibits limitations for large-scale production such as clogging when processing concentrated suspensions or when dealing with liposomes with substantially greater sizes than those of the membrane pores in the extruder [38]. Alternatively, no further processing is required with our devices (if a new one is used) due to continuous in-flow formation of nanovesicles of narrow size distributions controlled by the flow conditions [5,19].

### 3.6. Manufacturing Technique Benefits and Applications

Although the use of soft lithography and Polydimethylsiloxane (PDMS)as principal material promoted the widespread of the microfluidic technologies and has a relative low manufacturing cost compared to some techniques, such as the silicon etching and injection molding. It still requires a photomask and a microfabricated mold that limits the design optimization and represents a barrier for research and high-volume production [44,45]. Alternatively, the use of polymer sheets such as the PMMA has gained attention in recent years due to the possibility of creating structures by a wide range of techniques, which additionally offer better mechanical properties than the PDMS at a much lower cost [46]. For the specific case of the liposomes’ production by microfluidic approaches, several studies have reported on the successful application of hydrodynamic focusing (MHF) and passive micromixers for their synthesis in which the obtained sizes range from 20 nm to 308 nm with PDI values below 0.5 [5,6,30]. Nevertheless, in most cases, the preferred material is PDMS while the manufacturing technique is soft lithography [47,48,49]. In this study, we put forward a PMMA-based microfluidic device with a manufacturing cost below USD $2, which rely on widely available (and are currently declining in price) laser-cutting manufacturing instruments. Moreover, the assembly is conducted with simple and inexpensive gluing techniques that are also easily accessible in any lab. Our results showed that, despite the low-cost, the manufactured devices offer a robust platform for the reproducible and high-throughput production of nanoscale liposomes with acceptable physical properties, which are even comparable with those obtained by bulk methods and more expensive manufacturing cleanroom techniques. This opens the possibility for implementing our low-cost devices in different industrial and research applications involving liposomes due to the chances of achieving high encapsulation rates for different materials during their assembly process within the microfluidic device [50,51]. An interesting example for our research group is the encapsulation of cell-penetrating nano-vehicles. We hypothesized that the internalization potency of such nano-vehicles can be significantly improved by encapsulation into liposomes. This is critical to enable applications in drug delivery to overcome issues regarding limited passing across different biological barriers such as the cell membrane, the intestinal epithelium, and the blood brain barrier.

## 4. Conclusions

Liposomes have attracted significant attention as encapsulation vehicles for applications in several industries including cosmetics, pharma, and food. This can be attributed to their exceptional physicochemical properties that allow extending the lifetime of expensive bioactive compounds that otherwise might be compromised under physiological or even more extreme conditions. Despite the maturity of the technologies to produce liposomes and encapsulates with them, there is still challenges regarding their size distribution and stability. To address these issues, here, we proposed devices that allow the synthesis of liposomes with diameters in the nanoscale range and uniform particle size distributions. Additionally, the proposed manufacturing procedure is relatively simple and can be conducted without the use of sophisticated equipment or specialized facilities. The involved costs per device approached only USD $2. This is particularly attractive for laboratories operating under limited budgets as quality standards that are still maintained with low investments in equipment and materials compared with conventional clean room manufacturing processes. However, our approach still allows manufacturing of microfluidic systems with relatively complex channel geometries and dimensions with resolutions down to about 100 µm [26,52,53]. However, it is important to highlight that the operational half-life of the devices is limited mainly due to the presence of defects and imperfections on the microchannels as they favor the deposition of raw materials. This is detrimental to the performance of the device and, as time passes, it is possible to observe a marked decline in the homogeneity of the particle size distribution of the samples and the presence of impurities that might be able to induce coalescence processes.

The introduced manufacturing technique provides a promising route for low-cost prototyping of microfluidic devices where performance is not significantly compromised. We believe that this is valuable for running proof-of-concept experiments prior to conducting expensive high-resolution manufacturing.

## Figures and Tables

**Table 1:** Summary of results for the two-layers device.

Flow Rate	Aqueous to Lipid Solution Ratio	Average Size (nm)	PDI	Zeta Potential (mV)
5 mL/min (PBS)	2:1	250 ± 55	0.51 ± 0.13	−4.70 ± 1.53
	3:1	222 ± 43	0.22 ± 0.10	−10.13 ± 3.64
	4:1	191 ± 37	0.20 ± 0.08	−7.47 ± 2.15
	5:1	188 ± 61	0.32 ± 0.13	−11.65 ± 0.75
5 mL/min (NaCl)	2:1	462 ± 58	0.58 ± 0.03	−7.52 ± 1.10
	3:1	1312 ± 373	0.89 ± 0.03	−9.76 ± 2.70
	4:1	362 ± 10	0.22 ± 0.01	−13.11 ± 2.44
	5:1	283 ± 75	0.15 ± 0.03	−16.45 ± 2.93

**Table 2:** Summary of results for the three-layers device.

Flow Rate	Aqueous to Lipid Solution Ratio	Average Size (nm)	PDI	Zeta Potential (mV)
5 mL/min (PBS)	2:1	221 ± 63	0.39 ± 0.33	−4.91 ± 1.20
	3:1	520 ± 125	0.46 ± 0.16	−8.12 ± 1.70
	4:1	209 ± 50	0.18 ± 0.10	−14.11 ± 8.25
	5:1	345 ± 72	0.22 ± 0.14	−10.10 ± 2.80
5 mL/min (NaCl)	2:1	236 ± 102	0.42 ± 0.03	−7.50 ± 2.40
	3:1	487 ± 248	0.26 ± 0.13	−10.01 ± 5.04
	4:1	455 ± 28	0.27 ± 0.13	−12.50 ± 5.70
	5:1	224 ± 33	0.14 ± 0.02	−11.40 ± 3.00

**Table 3:** Summary of results for the synthesis of liposomes via traditional methods.

Method	Average Size (nm)	PDI	Zeta Potential (mV)
Freeze-Thaw	371 ± 76	0.34 ± 0.09	−12.81 ± 2.25
TFH	174 ± 0.450	0.21 ± 0.01	−10.45 ± 0.68

## Supplementary Materials

The following are available online at https://www.mdpi.com/2072-666X/11/12/1050/s1. Figure S1: dynamic light scattering (DLS) measurements of liposomes samples synthesized by changing the flow rate ratio (FRR) between an aqueous and solvent phase using the two-layer microfluidic device. (a) Two-layer device with PBS as an aqueous phase. (b) Two-layer device with NaCl solution (0.05 M) as an aqueous phase. Figure S2: (DLS)measurements of liposome samples synthesized by changing the flow rate ratio (FRR) between aqueous and solvent phase using the three-layer microfluidic device. A. The three-layer device with PBS solution as an aqueous phase. B. The three-layer device with NaCl solution (0.05 M) as an aqueous phase. Figure S3. DLS measurements. A. Freeze thaw method. B. Thin Hydration bilayer method. Each experiment was performed in triplicate (*n* = 3) and the analyses for liposome characterization were also performed in triplicate. This is indicated as the labeled replicas in Figure S1 and Figure S2. Table S1. Parameters of finite element method (FEM) simulation.
