# Supplementary material for: Synthesis of Nanoscale Liposomes via Low-Cost Microfluidic Systems
Source: Micromachines (Basel). 2020 Nov 28;11(12):1050. doi: 10.3390/mi11121050 (PMC7760644; doi:10.3390/mi11121050)
Supplement: Supplementary file 1 [file micromachines-11-01050-s001.pdf]

micromachines

Supplementary Materials

Synthesis of Nanoscale Liposomes via Low-Cost Microfluidic Systems

Andrés Aranguren [1,†], Carlos E. Torres [2,†], Carolina Muñoz-Camargo [2], Johann F. Osma [1,*] and Juan C. Cruz [2,3,*]

[1] Department of Electrical and Electronic Engineering, Universidad de los Andes, Cra. 1E No. 19a–40, Bogotá DC 111711, Colombia; a.aranguren@uniandes.edu.co
[2] Department of Biomedical Engineering, Universidad de los Andes, Cra. 1E No. 19a–40, Bogotá DC 111711, Colombia; ce.torres10@uniandes.edu.co (C.E.T.); c.munoz2016@uniandes.edu.co (C.M.-C.)
[3] School of Chemical Engineering and Advanced Materials, The University of Adelaide, South Australia 5005, Australia
* Correspondence: jf.osma43@uniandes.edu.co (J.F.O.); jc.cruz@uniandes.edu.co (J.C.C.)
† These authors contributed equally.

SI1. Dynamic light scattering (DLS) measurements of liposomes samples synthesized by the microfluidic system proposed

Each experiment was performed in triplicate (n = 3) and the analyses for liposome characterization were also performed in triplicate. This is indicated as the labeled replicas in Figure S1 and Figure S2

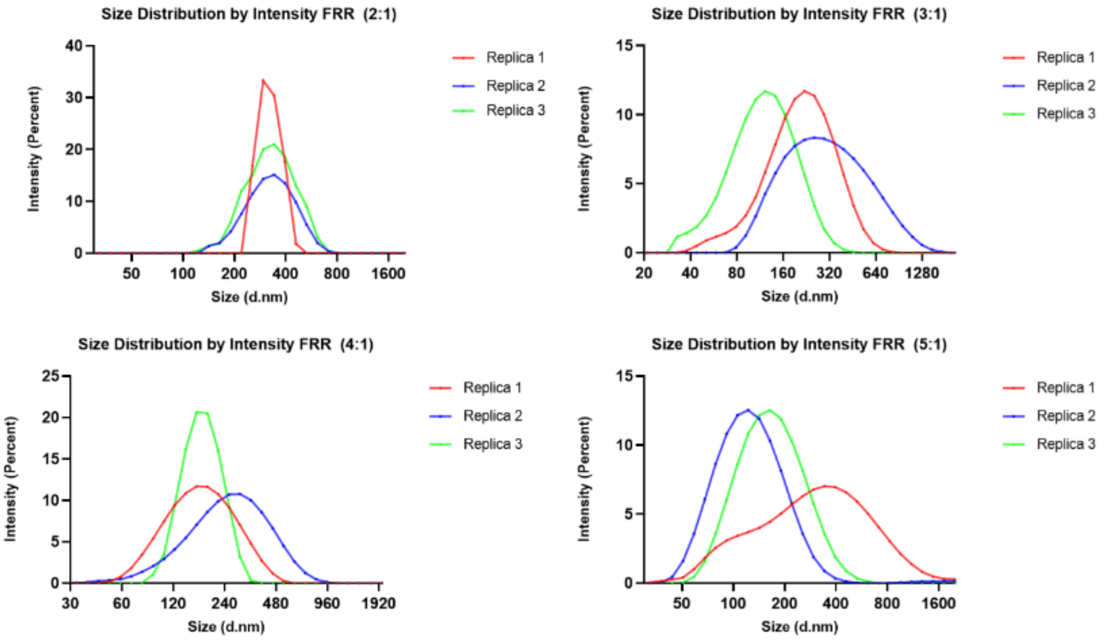

(a)

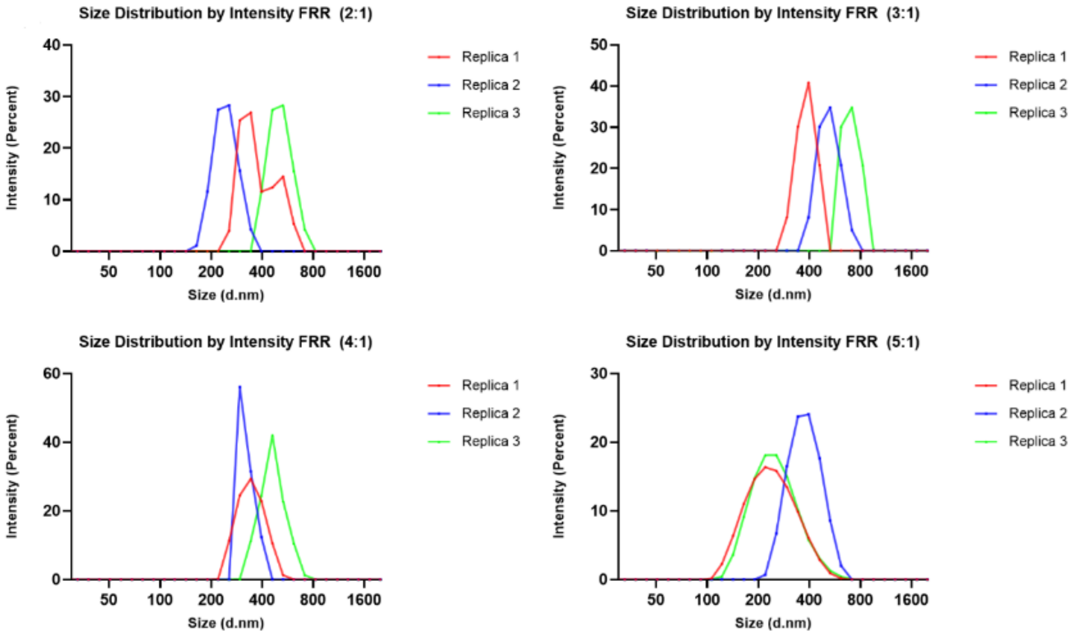

(b)

Figure S1. DLS measurements of liposomes samples synthesized by changing the flow rate ratio (FRR) between aqueous and solvent phase using the two-layer microfluidic device; (a) Two-layer device with PBS as aqueous phase; (b)Two-layer device with NaCl solution (0.05 M) as aqueous phase.

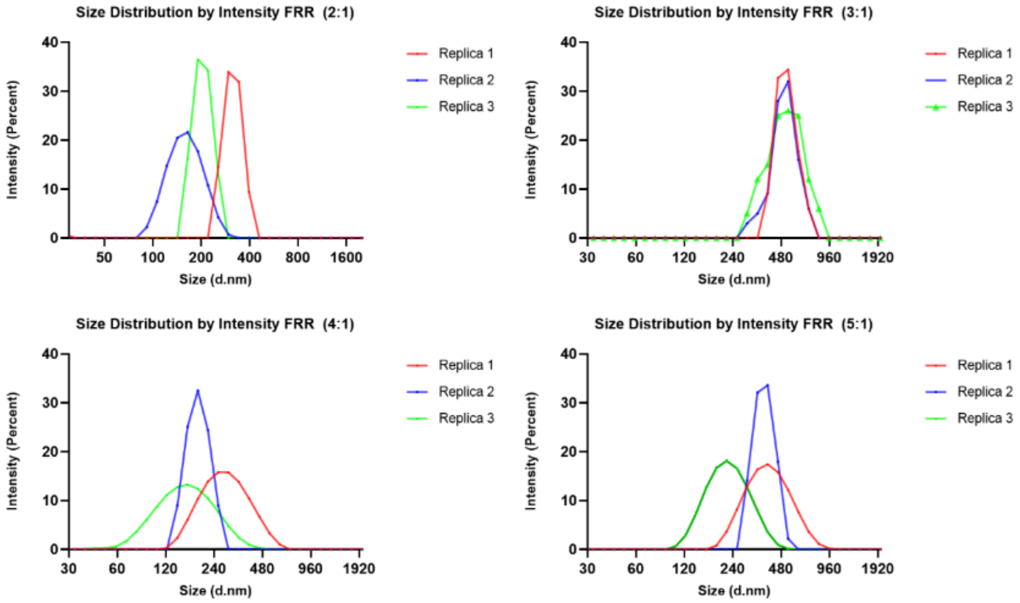

(a)

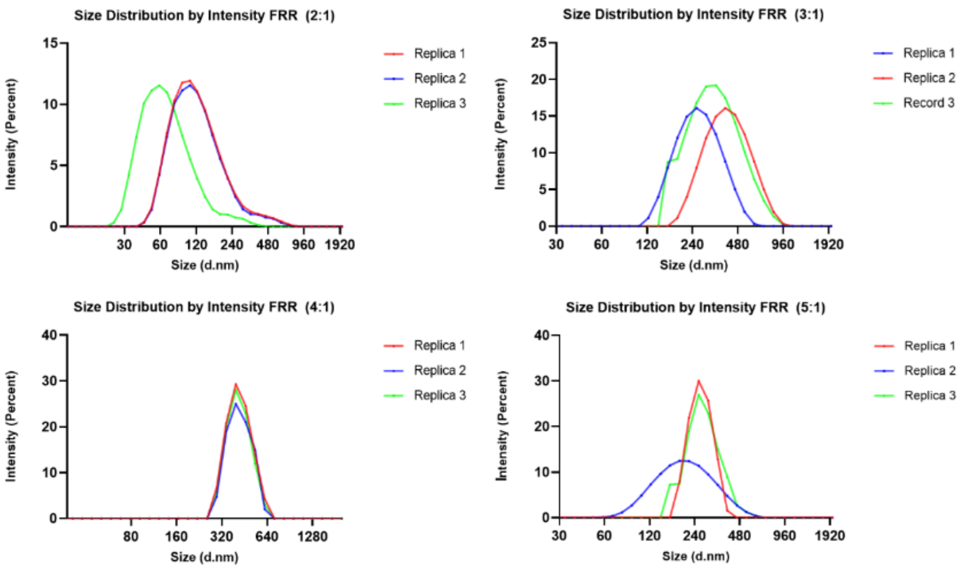

(b)

Figure S2. DLS measurements of liposomes samples synthesized by changing the flow rate ratio (FRR) between aqueous and solvent phase using the three-layer microfluidic device. A. Three-layer device with PBS solution as aqueous phase. B. Three-layer device with NaCl solution (0.05 M) as aqueous phase.

SI2. Dynamic light scattering (DLS) measurements of liposomes samples synthesized by the traditional methods

Each experiment was performed in triplicate (n = 3) and the analyses for liposome characterization were also performed in triplicate.

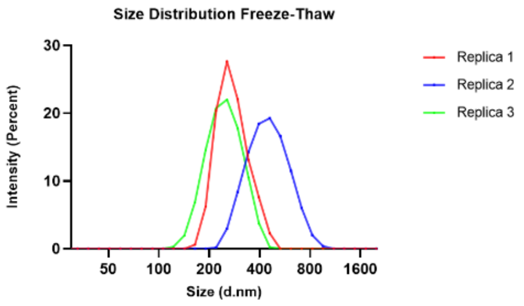

(a)

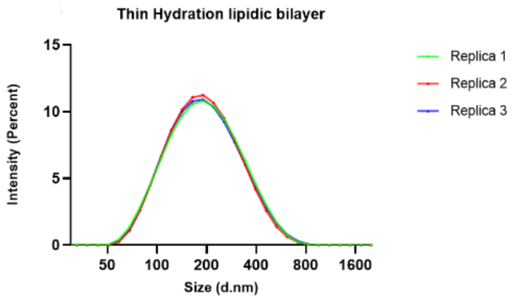

(b)

Figure S3. DLS measurements. A. Freeze thaw method. B. Thin Hydration bilayer method.

SI3. Parameters used in the simulations implemented in COMSOL Multiphysics®

The table S1 summarizes the parameters used in the Laminar flow and the transport of diluted species physics.

Table S1. Parameters of FEM simulation

Parameter	Value	Units
μ_{Water}	1.00E-3	Pa.s
ρ_{Water}	997	kg/m^3
μ_{IPA}	2.38E-3	Pa.s
ρ_{IPA}	783	kg/m^3
D_{LP}	1.64E-9	m^2/s

C_{LP}	1.73E6	mol/m^3

© 2020 by the authors. Licensee MDPI, Basel, Switzerland. This article is an open access article distributed under the terms and conditions of the Creative Commons Attribution (CC BY) license (http://creativecommons.org/licenses/by/4.0/).
